# Longest Known Survivor With HeartMate II Continuous‐Flow Left Ventricular Assist System—A Case Report of the 16‐Year Success Story

**DOI:** 10.1002/ccr3.9695

**Published:** 2025-01-06

**Authors:** Yamane Chawa, Mhd Baraa Habib, Ammar Chapra, Salah Elbdri

**Affiliations:** ^1^ Internal Medicine Department Hamad Medical Corporation Doha Qatar; ^2^ Cardiology Department, Heart Hospital Hamad Medical Corporation Doha Qatar

**Keywords:** destination therapy, heart failure left ventricular assistant device, HeartMate II, mechanical circulatory supportive device

## Abstract

Left ventricular assist devices (LVADs) have been used as a bridge to transplantation in patients with advanced heart failure. In this case, LVAD therapy was used as a destination therapy for 16 years, representing the longest documented and continuously ongoing support with the original implanted device.

**Abbreviations**
BT: bridging therapy
DT: destination therapy
INR: international normalized ratio
INTERMACS: Interagency Registry for Mechanically Assisted Circulatory Support
LVADs: left ventricular assist devices
MCS: mechanical circulatory support
NYHA: New York Heart Association
PCWP: pulmonary capillary wedge pressure
RVSP: right ventricular systolic pressure
STS: Society of Thoracic Surgeons

## 1 Introduction

Advanced heart failure significantly impacts a patient's survival, quality of life, and functional status. Heart transplantation is the optimal treatment for these patients, but challenges such as organ availability and suitability for transplantation exist [1]. Another option is long‐term inotropic therapy, but it is associated with a high mortality rate [2]. Mechanical circulatory support (MCS) devices, like the left ventricular assist device (LVAD), offer a breakthrough, restoring adequate organ perfusion and preventing organ dysfunction.

LVADs were initially developed as a “bridge to transplantation” or a short‐term solution for reversible causes. However, the REMATCH trial proposed that MCS could be a long‐term solution for patients unsuitable for heart transplantation [3]. This led to the use of LVADs therapy as destination therapy (DT) [2].

Initially, LVADs were pulsatile, but later studies showed that pulsatility wasn't mandatory for adequate support and end organ perfusion. This led to the creation of second‐generation LVADs, which were nonpulsatile or continuous flow [4]. The most successful among these was the HeartMate II, known for its durability because of its single moving part.

However, long‐term follow‐up with the HeartMate II as DT often results in complications because of the device malfunction or infection. We present a case of a patient who had the HeartMate II LVAD implanted successfully with a positive outcome.

## 2 Case Report

### 2.1 Case History

We present a clinical course of a 62‐year‐old Indian male patient with a medical background of type 2 diabetes mellitus, hypertension, and a long‐standing history of nonischemic dilated cardiomyopathy. Initially, the patient came to us with heart failure symptoms, which were categorized as class III according to the New York Heart Association (NYHA). Over the years, his systolic function progressively deteriorated, as evidenced by a drop in ejection fraction from 24% to 11% between 2001 and 2008, despite being on the highest dose of heart failure medication that he could tolerate. This decline necessitated the need for advanced heart failure treatment.

### 2.2 Initial Evaluation

The initial evaluation showed global biventricular hypokinesia, with severe LV dysfunction (left ventricular ejection fraction of 17.8%), cardiac output of 2.2L/min, and mild RV dysfunction. Other findings included a functioning tricuspid valve with minor regurgitation, an estimated pulmonary artery systolic pressure (RVSP) of 57 mmHg, and an estimated pulmonary capillary wedge pressure (PCWP) of 19.8 mmHg. The echo of the aortic valve showed a hardened but functioning tricuspid valve structure with a normal aortic root, arch, and descending aorta up to 35 cm from the incisors, without any thrombi or plaques. There was also a dilated mitral valve annulus with mild regurgitation and a central jet. Importantly, the coronary angiography revealed normal coronary arteries.

### 2.3 Intervention

The preoperative period was notable for frequent hospital admissions due to decompensated heart failure, classified as INTERMACS 4. Given that our institution did not have a heart transplantation program at that time, we decided to proceed with the implantation of an LVAD Heartmate II as a permanent solution. The surgery was successfully performed on August 28, 2008, followed by 10 days of close observation in the intensive care unit. A postoperative echocardiogram immediately confirmed proper LVAD function, showing a well‐functioning left ventricular inlet cannula with a peak pressure gradient of 1.00 m/sec. The aortic outlet cannula was also working effectively, with no residual aortic regurgitation, and the pericardium appeared normal during the evaluation. The patient spent an additional 20 days in the high‐dependency unit to optimize medical therapy and participate in the cardiac rehabilitation program. The hospital stay was uneventful, aside from the development of postoperative unilateral pleural effusion, which was successfully drained. Upon discharge, the patient’s INTERMACS status improved to 5, and he was prescribed warfarin with a target INR of 2‐3, alongside aspirin and the maximum tolerated doses of heart failure medication.

### 2.4 Complication

At postdischarge, the patient had several hospital admissions because of activated control alarms, which required the perfusionist's review and the controller device to be replaced twice. Additionally, the patient was once removed from his physical therapy session because of mild shortness of breath and palpitations, which led to the detection of nonsustained ventricular tachycardia (364 beats in run) and ventricular ectopic, which represent 5% of total QRS complexes on a 24‐h Holter study. Amiodarone was started and effectively managed the symptoms, as confirmed by a repeat Holter study. However, the patient developed amiodarone‐induced thyroiditis 3 years later, which necessitated the discontinuation of amiodarone and the start of a steroid regimen. Mexiletine was prescribed as an alternative medication.

### 2.5 Follow‐Up

The patient regularly visited our outpatient heart failure clinic every 3 months, which included laboratory tests and regular echocardiography examinations. These examinations consistently showed nonobstructive flow through the LVAD device, with a pressure gradient of 8 mmHg and a aortic valve opening with every beat, developing mild aortic insufficiency in his latest follow‐up. He demonstrated a strong commitment to the cardiac rehabilitation program, attending three sessions per week for 16 years, except during the COVID‐19 pandemic. However, he continued to exercise regularly from home during that time.

### 2.6 Long‐Term Care Planning

At a later stage, heart transplantation was considered, and the patient was willing to undergo the comprehensive workup for transplantation, which was completed. Unfortunately, because of various social barriers, the patient was unable to return to his home country for the transplant, leading to the decision to postpone the transplantation. However, it is worth mentioning that the patient is currently leading a satisfactory lifestyle 16 years after the LVAD insertion.

## 3 Discussion

The 13th annual report from The Society of Thoracic Surgeons (STS) INTERMACS highlights outcomes for 27,314 patients receiving continuous‐flow durable LVAD during the last decade (2012–2021). The last several years have been characterized by a shift in device indication and type, with 81.1% of patients now implanted as DT and 92.7% receiving an LVAD with full magnetic levitation in 2021 [4]. Which is relevant to our case, the change in decision of using LVAD as DT instead of bridging therapy (BT) after establishing an extreme durability continuing for 16 years with high cardiac support.

Even though devices such as HeartMate II have been studied for long‐term usage, and in our case the course of treatment went smoothly free from any major complications, but it is worth mentioning that the most common complications of CF LVADs include major bleeding, with GI bleeding being most common. Others include device malfunction, pump thrombosis, infections (whether due to LVAD device itself or otherwise), and stroke [5]. For HeartMate II, driveline and device infection are the most common reported adverse events according to one study, with sepsis and multisystem organ failure as the most common causes of death [6]. Another study found hemorrhagic stroke to be the most common cause of mortality in patients with HeartMate II. However, that may have been attributed to liberal anticoagulation protocols, which have since then been modified [2]. Despite reported complications, they were less frequently to occur with continuous flow LVADs when compared to pulsatile LVADs [2].

Case reports about patients surviving with HeartMate II for more than 10 years are limited for the usage of LVAD as DT, which emphasize their value for physicians as a long‐term solution. Even if patients survive with support, it may require repeated revision surgeries. Similarly, the longest DT survivor in the REMATCH trial had undergone as many as four device exchanges during the 7 years of LVAD support [3].

To contrast and compare our long‐term patient outcomes, we conducted a literature review (Table 1). This review aims to summarize insights from case reports, offering a comprehensive understanding of the outcomes, challenges, and successes in managing patients with HeartMate II LVADs. These cases of successful long‐term outcomes not only illuminate the adaptability of LVAD therapy but also highlight the importance of the collaborative efforts of a diverse team including cardiac surgeons, cardiologists, nurses, perfusionists, social workers, physiotherapists, and specifically LVAD coordinators, who play a vital role in patient care by preventing and promptly identifying LVAD‐related complications, as well as addressing patient‐specific risk factors and concerns to improve their quality of life.

**TABLE 1:** A literature review compiles essential findings from case reports, shedding light on the outcomes associated with managing patients who have undergone HeartMate II LVAD implantation.

Case	Author name	Patient characteristics	LVAD implantation	Complications	Survival
1 [7]	Francica, et al.	58‐year‐old man with situs viscerum inversus totalis (SVIT) and dextrocardia	2009	2 driveline infections (treated successfully with antibiotics), stable clinical conditions (NYHA class I‐II), good quality of life (KCCQ score of 79)	10‐year survival without device failure, thrombosis, or stroke
2 [8]	Letsou, et al.	46‐year‐old man with dilated aortic root, aortic insufficiency, and anomalous left main coronary artery	Not specified	Hepatic and renal failure, ileus, respiratory issues, LV wall issues, right ventricular failure, nonfunctional aortic valve, and thrombosis	Over a decade of LVAD functionality, despite complications
3 [9]	Kawabori, et al.	64‐year‐old man with hypertension, diabetes, renal insufficiency, ischemic dilated cardiomyopathy, previous CABG, and implanted cardioverter‐defibrillator	Not specified	Minimal complications, discharged after 46 days, survived over 500 days postoperatively	Survival over 500 days postoperatively, primary cause of death was respiratory failure because of aspiration pneumonia
4 [10]	Sundbom, et al.	31‐year‐old woman with left breast ductal carcinoma and subsequent heart failure due to anthracycline‐induced cardiotoxicity	November 2006	Minor driveline infections (successfully treated with antibiotics)	Almost 6 years of treatment with LVAD, followed by heart transplantation candidacy
5 [11]	Kristensen, et al.	80‐year‐old man with idiopathic dilated cardiomyopathy and atrial fibrillation since 30 years	2010	Recurrent bleeding from different organs including the gastrointestinal tract, skin, and brain. intestinal ischemia and underwent right hemicolectomy and ileostomy	14 years treatment with LVAD as DT
Our case	Chawa, et al.	62‐year‐old male with nonischemic cardiomyopathy	August 2008	Patient developed amiodarone induced thyroiditis after 3 years from using amiodarone to control episodes of ventricular tachycardia. Then switched to mexiletine	16 years treatment with LVAD as DT

Eventually, our case report presents a unique scenario of prolonged survival with the HeartMate II axial‐flow pump despite the generally superior outcomes demonstrated by the HeartMate 3 centrifugal‐flow pump in the MOMENTUM 3 trial [12]. This highlights the inherent variability in patient response to different treatment modalities and emphasizes the critical importance of individualized patient care. Although the MOMENTUM 3 trial offers valuable insights into device efficacy on a broader scale, it's essential to recognize and address the diverse needs of individual patients. We believe the key to our success lies in the patient's strong commitment to regular follow‐up appointments and strict adherence to cardiac rehabilitation over the years, as long‐term cardiac rehabilitation has demonstrated benefits for outpatients with LVADs, including better quality of life, decreased anxiety, and improved submaximal exercise capacity [13]. Further research is warranted to elucidate the factors influencing long‐term outcomes and to refine treatment strategies tailored to the specific needs of patients with LVADs.

## 4 Conclusion

This case underscores the pivotal role of LVADs as DT in patient's ineligible for heart transplantation because of either a shortage of donors or limited access to transplant centers. Our findings emphasize that LVAD therapy offers a viable alternative, significantly improving survival rates and enhancing quality of life in such patients. With its proven efficacy and ability to fill the gap where transplant options are limited, LVADs stand as a cornerstone in the management of advanced heart failure, providing hope and extending life for those in need. Finally, our case utilized HeartMate II and still had a favorable long‐term outcome. Considering the ongoing emergence of newer and improved LVADs such as HeartMate III, the prospects for this subset of patients are encouraging and will yield greater number of patients to be considered for LVAD as DT, especially as the technology continues to improve with a reduction in complications.

## Author Contributions

**Yamane Chawa:** supervision, writing – original draft, writing – review and editing. **Mhd Baraa Habib:** writing – original draft. **Ammar Chapra:** writing – original draft. **Salah Elbdri:** writing – review and editing.

## Ethics Statement

The case was approved for publication by Hamad Medical Corporation IRB.

## Consent

Written informed consent was obtained from the patient to publish this report in accordance with the journal's patient consent policy.

## Conflicts of Interest

The authors declare no conflicts of interest.

## Data Availability

Data sharing is not applicable to this article as no datasets were generated or analyzed during the current study.

## References

[1] S. A. Hunt , D. W. Baker , M. H. Chin , et al., “ACC/AHA Guidelines for the Evaluation and Management of Chronic Heart Failure in the Adult: Executive Summary A Report of the American College of Cardiology/American Heart Association Task Force on Practice Guidelines (Committee to Revise the 1995 Guidelines for the Evaluation and Management of Heart Failure): Developed in Collaboration With the International Society for Heart and Lung Transplantation; Endorsed by the Heart Failure Society of America,” Circulation 104, no. 24 (2001): 2996–3007, 10.1161/hc4901.102568. 11739319

[2] F. H. Sheikh and S. D. Russell , “HeartMate II Continuous‐Flow Left Ventricular Assist System,” Expert Review of Medical Devices 8, no. 1 (2011): 11–21, 10.1586/erd.10.77. 21158536

[3] E. A. Rose , A. J. Moskowitz , M. Packer , et al., “The REMATCH Trial: Rationale, Design, and End Points,” Annals of Thoracic Surgery 67, no. 3 (1999): 723–730, 10.1016/S0003-4975(99)00042-9. 10215217

[4] M. Yuzefpolskaya , S. E. Schroeder , B. A. Houston , et al., “The Society of Thoracic Surgeons Intermacs 2022 Annual Report: Focus on the 2018 Heart Transplant Allocation System,” Annals of Thoracic Surgery 115, no. 2 (2023): 311–327, 10.1016/j.athoracsur.2022.11.023. 36462544

[5] P. Shah , M. Yuzefpolskaya , G. W. Hickey , et al., “Twelfth Interagency Registry for Mechanically Assisted Circulatory Support Report: Readmissions After Left Ventricular Assist Device,” Annals of Thoracic Surgery 113, no. 3 (2022): 722–737, 10.1016/J.ATHORACSUR.2021.12.011. 35007505 PMC8854346

[6] J. S. Hanke , S. V. Rojas , C. Mahr , et al., “Five‐Year Results of Patients Supported by HeartMate II: Outcomes and Adverse Events,” European Journal of Cardio‐Thoracic Surgery 53, no. 2 (2018): 422–427, 10.1093/EJCTS/EZX313. 28958073

[7] A. Francica , F. Onorati , and G. Faggian , “Ten‐Year Survival With HeartMate‐II in Situs Viscerum Inversus,” Artificial Organs 45, no. 6 (2021): 639–640, 10.1111/aor.13882. 33474777

[8] G. V. Letsou , F. I. Musfee , A. D. Lee , F. Cheema , R. M. Delgado , and O. H. Frazier , “Ten‐Year Survival With a Continuous‐Flow Left Ventricular Assist Device and Aortic Valve Closure,” Texas Heart Institute Journal 47, no. 4 (2020): 325–328, 10.14503/THIJ-19-7193. 33472231 PMC7819440

[9] M. Kawabori , C. Kurihara , T. Sugiura , A. B. Civitello , and J. A. Morgan , “HeartMate II Implantation Technique That Spares the Sternum and Ascending Aorta,” Journal of Artificial Organs 21, no. 4 (2018): 458–461, 10.1007/s10047-018-1049-y. 29785544

[10] P. Sundbom , E. Hedayati , B. Peterzén , H. Granfeldt , H. Ahn , and L. Hubbert , “Young Woman With Breast Cancer and Cardiotoxicity With Severe Heart Failure Treated With a HeartMate IITM for Nearly 6 Years Before Heart Transplantation,” ASAIO Journal 60, no. 6 (2014): e3–e4, 10.1097/MAT.0000000000000138. 25232773

[11] A. Hallberg Kristensen , P. Svenarud , L. H. Lund , and E. Najjar , “The Longest Living Patient Supported With Left Ventricular Assist Device (14 Years),” ASAIO Journal 70 (2024): e147–e149, 10.1097/MAT.0000000000002184. 38446866

[12] M. R. Mehra , N. Uriel , Y. Naka , et al., “A Fully Magnetically Levitated Left Ventricular Assist Device—Final Report,” New England Journal of Medicine 380, no. 17 (2019): 1618–1627, 10.1056/NEJMoa1900486. 30883052

[13] T. Schlöglhofer , C. Gross , F. Moscato , et al., “Exercise Performance and Quality of Life of Left Ventricular Assist Device Patients After Long‐Term Outpatient Cardiac Rehabilitation,” Journal of Cardiopulmonary Rehabilitation and Prevention 43, no. 5 (2023): 346–353, 10.1097/HCR.0000000000000789. 37014949

